# A Madras Motor Neuron Disease Patient With Cerebellar Atrophy: A New Clinical Feature

**DOI:** 10.3389/fnins.2018.00722

**Published:** 2018-10-08

**Authors:** Ling Long, Xiaodong Cai, Jia Liu, Zhuang Kang, Jing Li, Zizhen Huang, Ruomi Guo, Yan Zou, Zhengqi Lu

**Affiliations:** ^1^Department of Neurology, Third Affiliated Hospital of Sun Yat-sen University, Guangzhou, China; ^2^Department of Neurology, Sixth Affiliated Hospital of Sun Yat-sen University, Guangzhou, China; ^3^Department of Radiology, Third Affiliated Hospital of Sun Yat-sen University, Guangzhou, China; ^4^Department of Otolaryngology, Third Affiliated Hospital of Sun Yat-sen University, Guangzhou, China

**Keywords:** MRI, cerebellum, atrophy, motor neuron disease, facial palsy

## Abstract

A 34-year-old Chinese Han female complaining of general muscle weakness and wasting for 9 years. She was admitted for aggravation of her symptoms caused by respiratory distress. She also suffered from bulbar palsy. She had no hearing loss, visual problems, or cerebellar signs. Her parents had a consanguineous marriage, though there was no family history of these symptoms. Pure tone audiometric findings demonstrated no definite abnormality. Electromyography demonstrated neurogenic damage. Brain magnetic resonance imaging revealed cerebellar atrophy, dominantly in anterior lobe. Gene sequencing of whole gene exomes was negative. She was finally diagnosed with Madras motor neuron disease (MMND), a rare subtype of motor neuron disease. No definite therapy was available for MMND, and she died of respiratory tract infection 1 year later. Previous studies have shown that cerebellar signs are positive in 17.2% patients of MMND, but no case with cerebellar atrophy has been reported before. Thus, here we describe cerebellar atrophy as a new clinical feature of MMND.

## Introduction

Madras motor neuron disease (MMND) was first described by Meenakshisundaram et al. (1970) in Madras in southern India. Patients usually present with onset at early age, atrophy, and weakness of the limbs, cranial nerve involvement, sensorineural hearing loss, and a slowly progressive disease course, though MMND does not seem to be benign in all patients (Nalini et al., 2006). MMND patients who have optic nerve atrophy have a variant of MMNDV (Gourie-Devi and Nalini, 2003). The majority of affected persons documented have been from southern India, and a few cases have been reported from outside India, such as in Korea (Im and Kim, 2008), Turkey (Isak et al., 2009), China (Fan et al., 2004; He et al., 2015), and other countries. Although China is geographically close to India, very few cases with MMND have been reported. Here, we presented an MMND case in southern China to further improve our understanding of this disease. This is the first case presenting with cerebellar atrophy.

## Case Report

In June 2016, a 34-year-old woman from Hunan Province in South China came to our hospital presenting with apparent weakness of the whole body, muscular atrophy, facial diplegia, hypophonia, dysphagia, and intermittent fasciculations of facial muscles starting in 2007 when she delivered her first child. The patient’s symptoms gradually deteriorated and worsened after her second child was born in 2011. She was diagnosed in local hospitals with gastritis at that stage. In 2012, she developed dysphagia and dysarthria. She had electromyography (EMG) in 2013, and the result showed extensive neurogenic damage, supported by reduced amplitude on musculus facialis, all limb muscles, and the sternocleidomastoid muscle. Two other hospitals diagnosed her with motor neuron disease (MND). She refused to use Riluzole, the first-line drug for amyotrophic lateral sclerosis (ALS), so no specific treatment was taken. She came to our hospital with respiratory distress, accompanied with symptoms of pneumonia such as cough and expectoration. She did not complain of visual or hearing impairment. She was a pharmacist and denied any poisonous substance exposure. Her father and mother had a first-degree consanguineous marriage. No other member in her family presented with any similar disorder.

**FIGURE 1:**
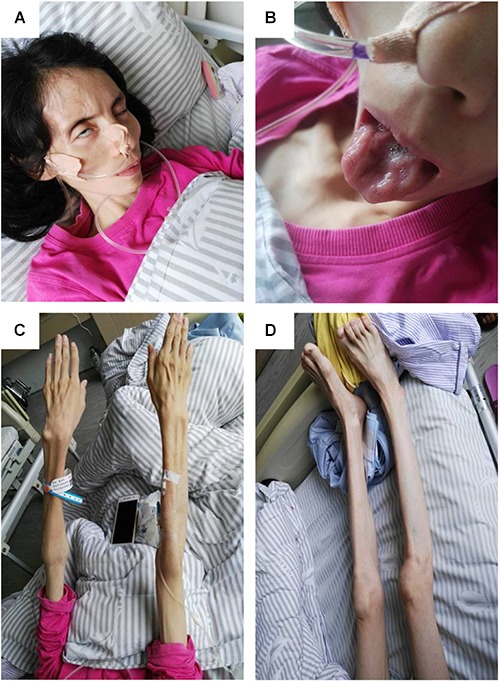
Pictures of the patient. **(A)** Obvious bilfacial weakness with obvious incomplete eyelid closure, which was more severe on the right side. **(B)** Severe tongue atrophy. **(C)** Symmetrical atrophy of upper limbs with involvement of the proximal and distal parts to the same extent. **(D)** Symmetrical atrophy of the lower limbs.

On physical examination, her higher mental function was found to be unaffected. Her height was 157 cm, and weight was 27 kg, with a body mass index of 10.95 kg/m^2^. She had facial diplegia (**Figure 1A**) with incomplete eyelid closure, and Bell sign was positive on the right side. Her muscles of mastication had decreased power and salivation was obvious. Sluggish palatal and gag reflexes, and fasciculation and atrophy of the tongue (**Figure 1B**) were observed. Fasciculation of the facial muscles was detected. She had generalized and symmetrical muscular atrophy, involving the face, trunk, and limbs (**Figures 1C,D**). She had an exhausted look with bulbar palsy and severe dyspnea. Muscle strength in the upper and lower limbs was assessed at United Kingdom Medical Research Council (MRC) grade IV. Bilateral Rossolimo sign was positive, and Babinsiki sign was positive on the right side. Tendon reflexes in the arms and legs were feeble. No sensory abnormality was detected. Coordinative movements including finger–nose incoordination, alternating movement, heel knee incoordination, and Romberg sign were normal. Meningeal irritation sign was absent.

After admission, the following tests were normal or negative: serum creatinine kinase, copper, mercury, anti-neutrophil cytoplasmic antibody, anti-nuclear antibodies, thyroid hormones and antibodies, HIV antibody, tumor markers, and electrocardiogram. Paraneoplastic antibodies including Hu, Yo, Ri, CV2, Ma2, Amphiphysin, TR, ANNA-3, PCA-2, and GAD were negative. Genetic studies for whole exome sequencing were negative, including *SOD1*, *ALS2*, *SETX*, *C9orf72*, and *FUS*.

Results of the following serum tests were abnormal (normal reference range in brackets): creatine 27 μmol/L (31.8–91.0 μmol/L), prealbumin 83 mg/L (200–400 mg/L), uric acid 74 μmol/L (90–420 μmol/L), low density lipoprotein 1.93 mmol/L (2.07–3.10 μmol/L), apolipoprotein B 0.52 g/L (0.6–1.1 g/L), cysteine protease inhibitor 0.39 mg/L (0.55–1.55 mg/L), β2 microglobulin 0.72 mg/L (1.00–3.00 mg/L), serum iron 2.1 μmol/L (11.0–27.0 μmol/L), and zinc 9.64 μmol/L (11.1–19.4 μmol/L). Superoxide dismutase (SOD) 121 U/ml (129–216 U/ml), vitamin B12 184 pg/ml (200–900 pg/ml), and vitamin B2 189.7 μg/L (>200 μg/L).

Her blood white cell count was 10.8 × 10^E9^/L, neutrophil rate 87.4% (40–75%). Arterial blood gas analysis showed her oxygen was 72 mmHg and carbon dioxide 57 mmHg, and lactic acid 0.8 mmol/L, while the pH was normal. Sputum smear found gram positive cocci and *Candida albicans*, but sputum cultures for bacteria and fungus were negative. Abdomen ultrasound revealed hepatic microhemangioma.

**FIGURE 2:**
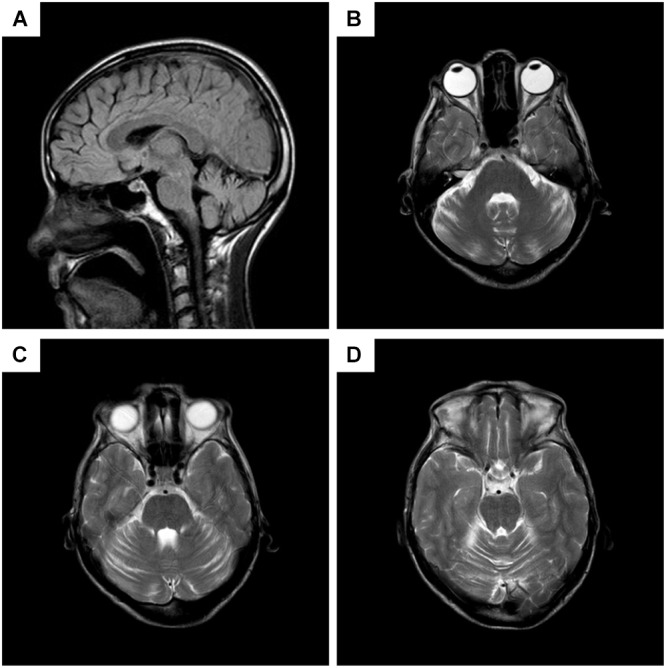
Brain MRI of the patient. **(A)** Sagittal T2 fluid attenuated inversion recovery (FLAIR) imaging, showing decreased volume of the anterior lobe of cerebellum, while cerebral lobes, periventricular white matters, and the brain stem are not atrophied. **(B–D)** Different planes of axial T2 imaging, presenting slim cerebellar gyrus and deepened cerebellar sulcus in anterior lobe.

Chest CT showed bilateral pneumonia. Mild reduction in low frequencies (125, 250, and 500 Hz) in the left ear was detected by pure tone audiometry. EMG showed denervation in the muscles of the right paravertebral T10 and lower limbs, and chronic reinnervation in the upper and lower limbs. The patient refused EMG of the sternocleidomastoid muscle. Brain MRI demonstrated atrophy of cerebellum, with anterior lobe involved more severely (**Figure 2**).

Our patient was characterized with progressive general muscle weakness and atrophy, involving the limbs, trunk, and muscles of cranial nerves (cranial nerve VII, X, and XI). Clinical signs and symptoms and EMG suggested damage to the pyramidal tract, anterior horn cells, and bulbar nuclei. Her history and laboratory findings excluded intoxication, metabolism disorders, vascular diseases, trauma, tumors, and autoimmune diseases. The consanguineous parents suggested that it might be an inherited disorder, though exome investigations were normal. Her condition was special in the involvement of the facial nerve, which is rare in MND. After analysis, her clinical manifestations indicated MMND. So, the final diagnosis of MMND, with complications of pneumonia, respiratory failure (type 2), and malnutrition, was made. We treated her with intravenous immunoglobulin (IVIG) (0.4 g/kg once daily for 5 days) and other symptomatic treatment (e.g., oxygen inhalation, intravenous levofloxacin, and nutritional support). Her symptoms of cough and expectoration relieved a little, but her other symptoms did not improve. She refused ventilatory support by continuous positive airway pressure. She was discharged after the pneumonia was controlled, but intermittently needed treatment for pneumonia and respiratory failure. Her condition deteriorated and she died of respiratory tract obstruction caused by sputum in March 2017. We got her husband’s inform consent for reporting this case.

## Discussion

Madras motor neuron disease is a special type of MND, constituting 0.9%–3.7% of all patients with MND (Gourie-Devi and Suresh, 1988; Saha et al., 1997). Common striking features of MMND are young age at onset, sporadic occurrence, sensorineural hearing loss, multiple lower cranial nerve palsy, diffuse atrophy with weakness of limbs, and a progressive but benign course (Gourie-Devi and Suresh, 1988). Although some features of MMND are like ALS, the onset age, facial palsy, and deafness distinguished them. Some cases have optic atrophy and cerebellar involvement, and MMND with optic atrophy was called MMNDV (Gourie-Devi and Nalini, 2003; Nalini et al., 2008). Our patient did not present with optic atrophy. Cerebellar signs were found in 20 of 116 patients (17.2%) in Nalini’s study (Nalini et al., 2008). Though coordinative motor function was preserved in our patient, cerebellar atrophy was identified on MRI. Given the negative family history and exome sequencing results, we consider our patient as a sporadic case. MMND has an unique geographical distribution with concentration of the majority of cases in southern India, and there were isolated reports of patients with MMND from Korea (Im and Kim, 2008), Turkey (Isak et al., 2009), China (Fan et al., 2004; He et al., 2015), Pakistan (Khan and Rashid, 2013), Thailand (Phanthumchinda et al., 1996), and Italy (who is of South Indian origin) (Massa et al., 1998). Our patient was the fifth case in China, without any Indian blood relationship.

The pathogenesis of the disease is unknown. Although most of the reported cases are apparently sporadic (Massa et al., 1998), there are reports of familial MMND (FMMND), furthermore, and 13.8% cases (including non-familial and familial MMND) have parent consanguinity (Nalini et al., 2008), so genetic changes should be taken into account. In some family groups, there is a possibility of autosomal dominant or autosomal recessive inheritance (Nalini et al., 2006). Higher numbers of sporadic cases also suggest the possibility of *de novo* rearrangements of an autosomal recessive gene (Nalini et al., 2008). However, no pathogenic genes have been reported. We did not find any changes in our patient’s exome. Mitochondrial DNA variations may have an indirect role in the pathogenesis of MMND (Govindaraj et al., 2013), but we did not check mitochondrial DNA in this case. Additionally, Valmikinathan et al. (1973) suggested that the pathophysiology of MMND might be associated with altered citrate metabolism. The first biopsy proposed that the possibility of an inflammatory etiology for MMND needs to be considered (Phanthumchinda et al., 1996). Serum SOD level in our patient was mildly decreased, supporting the hypothesis that inflammation might partially contribute to MMND. Though cerebellar signs were not obvious in our patient, MRI revealed cerebellar atrophy, suggesting neurodegeneration might play a key role.

Like other MNDs, no definitive treatment is available. IVIG might play a role in improving symptoms (Fan et al., 2004). However, our patient did not achieve a remarkable improvement from IVIG. MMND has a slower progression with a relatively longer survival period compared with ALS. Nevertheless, there were patients who indeed had a short survival period. Women appeared to have a shorter survival period as compare to men.

Although cerebellar signs are common in MMND, appearing in 17.2% cases in Nalini et al.’s (2008) study, brain CT and MRI presented normally in previous studies. Our patient did not behave obvious cerebellar signs such as dysarthria, ataxia, and nystagmus, however, MRI images showed cerebellar atrophy in anterior lobe. So our case is the first to show that cerebellar atrophy can exist in MMND.

## Concluding Remarks

Madras motor neuron disease is a rare disorder, so it is unfamiliar to most clinicians. It may be easily overlooked, and clinical diagnosis may be delayed. Understanding of the disease should be deepened to facilitate neurologists’ recognition of it. Further investigations of mechanisms should be conducted to help with the development of treatments. Brain imaging of such cases have not been well described before. Here, we have presented the first case with cerebellar atrophy, and more imaging studies are needed to further describe it.

## Availability of Data and Supplementary Materials

Data available from the authors upon request.

## Ethics Statement

A written informed consent was obtained from the patient’s husband (because the patient died) for the publication of this case report. No investigation or intervention was performed outside routine clinical care for this patient. As this is a case report, without experimental intervention into routine care, no formal research ethics approval is required.

## Author Contributions

LL, XC, and JLiu wrote the manuscript. ZK, RG, and YZ analyzed MRI results. JLi analyzed electromyography results. ZH analyzed pure tone audiometric findings. YZ and ZL analyzed the data. All authors made a critical revision of the manuscript.

## Conflict of Interest Statement

The authors declare that the research was conducted in the absence of any commercial or financial relationships that could be construed as a potential conflict of interest.
